# Supplementary material for: HIVprotI: an integrated web based platform for prediction and design of HIV proteins inhibitors
Source: J Cheminform. 2018 Mar 9;10:12. doi: 10.1186/s13321-018-0266-y (PMC5845081; doi:10.1186/s13321-018-0266-y)
Supplement: Supplementary file 2 — Additional file 2. Source code of HIVProtI web server. [file 13321_2018_266_MOESM2_ESM.zip › HIVprotI_Source-code/hivprotI/weka-3-6-6/doc/weka/core/ClassDiscovery.html]

ClassDiscovery


---


|  |  |  |
| --- | --- | --- |
| **Overview** \| **Package** \| **Class** \| **Tree** \| **Deprecated** \| **Index** \| **Help** \| **Weka's home** | |  |
| **PREV CLASS**   **NEXT CLASS** | **FRAMES**    **NO FRAMES**     **All Classes** |  |
| SUMMARY: NESTED \| FIELD \| CONSTR \| METHOD | DETAIL: FIELD \| CONSTR \| METHOD |  |


---


## weka.core Class ClassDiscovery

```
java.lang.Object
  weka.core.ClassDiscovery
```

**All Implemented Interfaces:**: RevisionHandler

---

``` public class ClassDiscovery extends java.lang.Object implements RevisionHandler ```

This class is used for discovering classes that implement a certain
interface or a derived from a certain class.

**Version:**
:   $Revision: 5377 $

**Author:**
:   FracPete (fracpete at waikato dot ac dot nz)

**See Also:**: `ClassDiscovery.StringCompare`

---

| **Nested Class Summary** | |
| --- | --- |
| `static class` | `ClassDiscovery.StringCompare`             compares two strings. |


| **Field Summary** | |
| --- | --- |
| `static boolean` | `VERBOSE`             whether to output some debug information. |


**Constructor Summary**

`ClassDiscovery()`


| **Method Summary** | |
| --- | --- |
| `static void` | `clearCache()`             clears the cache for class/classnames relation. |
| `static java.util.Vector` | `find(java.lang.Class cls, java.lang.String pkgname)`             Checks the given package for classes that inherited from the given class, in case it's a class, or implement this class, in case it's an interface. |
| `static java.util.Vector` | `find(java.lang.Class cls, java.lang.String[] pkgnames)`             Checks the given packages for classes that inherited from the given class, in case it's a class, or implement this class, in case it's an interface. |
| `static java.util.Vector` | `find(java.lang.String classname, java.lang.String pkgname)`             Checks the given package for classes that inherited from the given class, in case it's a class, or implement this class, in case it's an interface. |
| `static java.util.Vector` | `find(java.lang.String classname, java.lang.String[] pkgnames)`             Checks the given packages for classes that inherited from the given class, in case it's a class, or implement this class, in case it's an interface. |
| `static java.util.Vector` | `findPackages()`             Lists all packages it can find in the classpath. |
| `java.lang.String` | `getRevision()`             Returns the revision string. |
| `static boolean` | `hasInterface(java.lang.Class intf, java.lang.Class cls)`             Checks whether the given class implements the given interface. |
| `static boolean` | `hasInterface(java.lang.String intf, java.lang.String cls)`             Checks whether the given class implements the given interface. |
| `static boolean` | `isSubclass(java.lang.Class superclass, java.lang.Class otherclass)`             Checks whether the "otherclass" is a subclass of the given "superclass". |
| `static boolean` | `isSubclass(java.lang.String superclass, java.lang.String otherclass)`             Checks whether the "otherclass" is a subclass of the given "superclass". |
| `static void` | `main(java.lang.String[] args)`             Possible calls: weka.core.ClassDiscovery <packages> |

**Methods inherited from class java.lang.Object**

`equals, getClass, hashCode, notify, notifyAll, toString, wait, wait, wait`

**Field Detail**

### VERBOSE

```
public static final boolean VERBOSE
```

:   whether to output some debug information.

    **See Also:**: Constant Field Values


**Constructor Detail**

### ClassDiscovery

```
public ClassDiscovery()
```


**Method Detail**

### isSubclass

```
public static boolean isSubclass(java.lang.String superclass,
                                 java.lang.String otherclass)
```

:   Checks whether the "otherclass" is a subclass of the given "superclass".

    :   **Parameters:**: `superclass` - the superclass to check against: `otherclass` - this class is checked whether it is a subclass of the the superclass **Returns:**: TRUE if "otherclass" is a true subclass

---


### isSubclass

```
public static boolean isSubclass(java.lang.Class superclass,
                                 java.lang.Class otherclass)
```

:   Checks whether the "otherclass" is a subclass of the given "superclass".

    :   **Parameters:**: `superclass` - the superclass to check against: `otherclass` - this class is checked whether it is a subclass of the the superclass **Returns:**: TRUE if "otherclass" is a true subclass

---


### hasInterface

```
public static boolean hasInterface(java.lang.String intf,
                                   java.lang.String cls)
```

:   Checks whether the given class implements the given interface.

    :   **Parameters:**: `intf` - the interface to look for in the given class: `cls` - the class to check for the interface **Returns:**: TRUE if the class contains the interface

---


### hasInterface

```
public static boolean hasInterface(java.lang.Class intf,
                                   java.lang.Class cls)
```

:   Checks whether the given class implements the given interface.

    :   **Parameters:**: `intf` - the interface to look for in the given class: `cls` - the class to check for the interface **Returns:**: TRUE if the class contains the interface

---


### find

```
public static java.util.Vector find(java.lang.String classname,
                                    java.lang.String[] pkgnames)
```

:   Checks the given packages for classes that inherited from the given class,
    in case it's a class, or implement this class, in case it's an interface.

    :   **Parameters:**: `classname` - the class/interface to look for: `pkgnames` - the packages to search in **Returns:**: a list with all the found classnames

---


### find

```
public static java.util.Vector find(java.lang.String classname,
                                    java.lang.String pkgname)
```

:   Checks the given package for classes that inherited from the given class,
    in case it's a class, or implement this class, in case it's an interface.

    :   **Parameters:**: `classname` - the class/interface to look for: `pkgname` - the package to search in **Returns:**: a list with all the found classnames

---


### find

```
public static java.util.Vector find(java.lang.Class cls,
                                    java.lang.String[] pkgnames)
```

:   Checks the given packages for classes that inherited from the given class,
    in case it's a class, or implement this class, in case it's an interface.

    :   **Parameters:**: `cls` - the class/interface to look for: `pkgnames` - the packages to search in **Returns:**: a list with all the found classnames

---


### find

```
public static java.util.Vector find(java.lang.Class cls,
                                    java.lang.String pkgname)
```

:   Checks the given package for classes that inherited from the given class,
    in case it's a class, or implement this class, in case it's an interface.

    :   **Parameters:**: `cls` - the class/interface to look for: `pkgname` - the package to search in **Returns:**: a list with all the found classnames

---


### findPackages

```
public static java.util.Vector findPackages()
```

:   Lists all packages it can find in the classpath.

    :   **Returns:**: a list with all the found packages

---


### clearCache

```
public static void clearCache()
```

:   clears the cache for class/classnames relation.

---


### getRevision

```
public java.lang.String getRevision()
```

:   Returns the revision string.

    :   **Specified by:**: `getRevision` in interface `RevisionHandler`
    :   **Returns:**: the revision

---


### main

```
public static void main(java.lang.String[] args)
```

:   Possible calls:

    - weka.core.ClassDiscovery <packages>
    - weka.core.ClassDiscovery <classname> <packagename(s)>

    :   **Parameters:**: `args` - the commandline arguments


---


|  |  |  |
| --- | --- | --- |
| **Overview** \| **Package** \| **Class** \| **Tree** \| **Deprecated** \| **Index** \| **Help** \| **Weka's home** | |  |
| **PREV CLASS**   **NEXT CLASS** | **FRAMES**    **NO FRAMES**     **All Classes** |  |
| SUMMARY: NESTED \| FIELD \| CONSTR \| METHOD | DETAIL: FIELD \| CONSTR \| METHOD |  |


---
